# Epidemiologic features, clinical characteristics, and predictors of mortality in patients with candidemia in Alameda County, California; a 2017–2020 retrospective analysis

**DOI:** 10.1186/s12879-022-07848-8

**Published:** 2022-11-12

**Authors:** Didien Meyahnwi, Bekure B. Siraw, Arthur Reingold

**Affiliations:** 1 School of Public Health, University of California, Berkeley, Berkeley, CA USA; 2 Division of Epidemiology, School of Public Health, University of California, Berkeley, Berkeley, CA USA

**Keywords:** Candidemia, Invasive candidiasis, Emerging infections, Bloodstream infections, Candida

## Abstract

**Background:**

Bloodstream infections caused by *Candida* species are responsible for significant morbidity and mortality worldwide, with an ever-changing epidemiology. We conducted this study to assess trends in the epidemiologic features, risk factors and *Candida* species distribution in candidemia patients in Alameda County, California.

**Methods:**

We analyzed data collected from patients in Alameda County, California between 2017 and 2020 as part of the California Emerging Infections Program (CEIP). This is a laboratory-based, active surveillance program for candidemia. In our study, we included incident cases only.

**Results:**

During the 4-year period from January 1st, 2017, to December 31st, 2020, 392 incident cases of candidemia were identified. The mean crude annual cumulative incidence was 5.9 cases per 100,000 inhabitants (range 5.0–6.5 cases per 100,000 population). *Candida glabrata* was the most common *Candida* species and was present as the only *Candida* species in 149 cases (38.0%), followed by *Candida albicans*, 130 (33.2%). Mixed *Candida* species were present in 13 patients (3.3%). Most of the cases of candidemia occurred in individuals with one or more underlying conditions. Multivariate regression models showed that age ≥ 65 years (RR 1.66, CI 1.28–2.14), prior administration of systemic antibiotic therapy, (RR 1.84, CI 1.06–3.17), cirrhosis of the liver, (RR 2.01, CI 1.51–2.68), and prior admission to the ICU (RR1.82, CI 1.36–2.43) were significant predictors of mortality.

**Conclusions:**

Non-albicans *Candida* species currently account for the majority of candidemia cases in Alameda County.

**Supplementary Information:**

The online version contains supplementary material available at 10.1186/s12879-022-07848-8.

## Introduction

*Candida* species are an important cause of bloodstream infections (BSIs) and are the leading cause of invasive fungal infections in hospitalized patients in the U.S [1]. Invasive candidiasis includes, among other manifestations, intra-abdominal infections, osteomyelitis, and bloodstream infections i.e., candidemia. In the United States and elsewhere, *Candida* species are a leading cause of health care–associated bloodstream infections [2]. Candidemia is associated with substantial morbidity, mortality, and increased healthcare costs [3]. The crude mortality rate among patients with candidemia is in the range of 40–75% [4]. Despite the fact that *Candida albicans* is still considered the most common cause of candidemia worldwide, a shift to non-albicans *Candida* (NAC) species has been observed globally in recent studies [5]. For instance, Ma et al., after reviewing candidemia data from a tertiary care hospital in China from 2009 to 2011, reported that the most frequent *Candida* spp. involved was *C. tropicalis* (28.6%), followed by *C. albicans* (23.3%) and *C. parapsilosis* (19.5%) [6]. Furthermore, the ARTEMIS DISK Global Antifungal Surveillance Study, a wide-ranging study had similar findings, while highlighting the variation in the distribution of *Candida* spp. isolates by geographical location [5].

Data from this registry showed that only five species (*C. albicans*, *C. glabrata*, *C. tropicalis*, *C. parapsilosis*, and *C. krusei*) collectively accounted for 92% of cases of candidemia [7]. Even though *C. albicans* was the most common cause of candidemia worldwide, considerable differences were found in the proportions of cases caused by *C. glabrata* and *C. parapsilosis.* Studies from Northern Europe and the USA reported a high proportion of cases caused by *C. glabrata* and a low proportion of cases caused by *C. parapsilosis*. In contrast, reports from Spain and Brazil demonstrated a lower proportion of cases caused by *C. glabrata* and a higher proportion of cases attributed to *C. parapsilosis *[5]*.* The explanation for these differences is unknown, although it may be a consequence of the impact of climate, policies concerning the use of antifungal drugs, and central venous catheter care procedures.

It is possible that the observed shift from *Candida* albicans to NAC species is due to the modifications in clinical practices that have gradually selected for NAC. Over the last two decades, for instance, new antifungal drugs and new management strategies, such as use of antifungal drugs as prophylaxis and pre-emptive therapy using triazoles or echinocandins, have been recommended in high-risk hospital patient populations, in particular patients with hematological malignancies and critically ill patients [8]. Their more frequent use may have influenced the *Candida* spp. distribution and antifungal susceptibility patterns [9].

Though the *Candida* species distribution has varied, several studies have reported that conditions such as diabetes mellitus, hematologic or solid organ malignancy, chronic kidney disease, intensive care unit (ICU) admission, among others, are common in most candidemia patients [6, 10–12]. However, risk factors for mortality in patients with candidemia have varied across studies. Xiao et al., noted that increasing age, decreased mean arterial pressure (MAP), and low Glasgow Coma Scale score (GCS), were independently associated with mortality in candidemia patients in China [13]. Meanwhile, Francesco et al., reported that older age, ICU admission, a recent diagnosis of cardiovascular disease and lack of an early central venous catheter removal were all associated with a significantly higher probability of death [11].

Given the substantial morbidity and mortality associated with candidemia, the observed geographic variation in *Candida* species distribution, and corresponding variation in antifungal drug susceptibility, we therefore examined the epidemiologic features of candidemia in Alameda County, California over the 4-year interval from 2017 to 2020 and determined the factors independently associated with mortality in such patients. The resulting data can help guide health professionals to tailor empiric antifungal therapy to the most likely involved *Candida* species.

## Methods

### Study design and data source

We conducted a retrospective cohort study using data collected between 2017 and 2020 by the California Emerging Infections Program (CEIP) on candidemia cases in Alameda County, California (Additional files 1, 2). The CEIP candidemia surveillance is an active, laboratory-based surveillance program that identifies all culture-confirmed candidemia in Alameda County hospitals [14].

### Study site

Alameda County is one of the nine counties of the San Francisco Bay area. According to the US decennial census of 2020, it had an estimated population of about 1,682,353 residents [15]. Alameda County has a “dry-summer subtropical” climate, often referred to as a Mediterranean climate. The average temperature for the year in Alameda is 15.7 °C.

### Surveillance method and case definition

In the event of laboratory confirmation of a case of candidemia in Alameda County, surveillance officers collect patient information from medical records using standardized data collection forms. Fungal isolates from the initial blood cultures are sent to the US Centers for Disease Control and Prevention (CDC) for further confirmation and speciation. The species identification was performed using the matrix-assisted laser desorption/ionization time of flight (MALDI-TOF) method [14].

### Case definition

An incident case of candidemia is defined as the first isolation of *Candida spp*. from the blood of a resident of Alameda County on or after January 1, 2017 [14].

Recurrent episodes: Candidemia cases that have a positive blood culture for *Candida* spp. > 30 days from the initial positive culture are considered a new case [14].

Subsequent cultures: Candidemia cases that have a positive blood culture for *Candida* spp. < 30 days from the initial positive culture are considered a subsequent case [14].

### Inclusion criteria

For the purposes of this study, we included all incident cases of candidemia in the CEIP database with positive culture dates from January 1, 2017, to December 31, 2020.

### Exclusion criteria

All positive cases reported to the CEIP between January 1, 2017, and December 31, 2020, which were cases with a sample collection day that predates January 1, 2017, and duplicate entries were excluded. All subsequent cases of candidemia in Alameda between 2017 and 2020 were also excluded.

### Statistical analysis

Crude candidemia incidence rates per 100,000 population were calculated by year. Percentages, age, sex, and race-specific incidence rates were calculated for various demographic subgroups. The race-specific incidence rates were calculated to explore incidence of candidemia with a health equity focus. The denominators utilized in calculating the incidence rates for the various demographic subgroups were obtained from the U.S. Census Bureau population and housing unit estimates for each corresponding year [15]. The count data were described by case number (n). Chi-square tests were used to assess the differences in proportions between two groups. Bivariate and multivariate modified Poisson regression models with robust estimators were used to identify risk factors independently associated with mortality, as described by Zou et al., for estimating relative risks in regression models with common outcomes using the ‘geepack’ package [16]. For the multivariate regression analysis, variables considered as candidates were those believed a priori to be clinically significant; those that had been identified in a previous study; and those significantly associated with mortality in the bivariate analysis. These variables include age, hospitalization 90 days before positive culture, underlying chronic conditions, total parenteral nutrition, pancreatitis, presence of a central venous catheter, neutropenia, systemic antifungals 14 days before culture, systemic antibiotic therapy 14 days before culture, ICU admission 14 days before culture, surgery 90 days before culture, neutropenia, presence of a hematological malignancy, presence of a solid organ malignancy, HIV infection, pregnancy, obesity. The multivariate analysis was conducted using stepwise procedures with different subsets of data. All tests were conducted at a significance level of α = 0.05. Data analysis was done using R statistical software (version 3.6.2).

### Ethical clearance

The CEIP acquired Ethical clearance for the collection of patient data for its surveillance program.

## Results

### Demographic characteristics and crude incidence rates

**Fig. 1:**
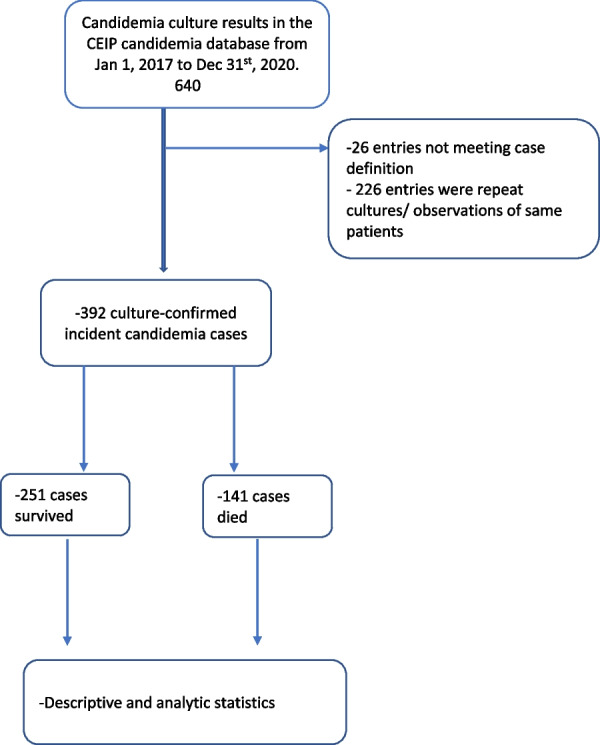
Flow chart of data collection, data cleaning, and analysis

From January 1, 2017, to December 31, 2020, 392 incident cases of candidemia were identified in residents of Alameda County (Fig. 1). The median age of the patients was 62 years (interquartile range, 52–74 years). Almost half (47.0%) of the patients were ≥ 65 years old. Seven (1.8%) of the patients were < 18 years of age and they made up the smallest fraction of the study population (Table 1). There were more males than females in the population (229 vs 163), representing 58.4% vs 41.6% respectively. Most of the patients were white (132, 33.7%), followed by Black or African American (100, 25.5%).

**Table 1:** Demographic characteristics of patients with candidemia in Alameda County, 2017–2020

	Level	Overall	2017	2018	2019	2020
Number		392	83	108	97	104
Sex (%)	Male	229 (58)	47 (57)	60 (56)	60 (62)	62 (60)
	Female	163 (42)	36 (43)	48 (44)	37 (38)	42 (40)
Age in years^a^ (%)	0–17	7 (2)	1 (1)	3 (3)	1 (1)	2 (2)
	18–44	65 (17)	14 (17)	20 (19)	18 (19)	13 (13)
	45–64	132 (34)	31 (37)	30 (29)	33 (34)	38 (38)
	≥ 65	181 (47)	37 (45)	52 (50)	44 (46)	48 (48)
Ethnicity (%)	Hispanic	64 (20)	17 (27)	13 (14)	18 (23)	16 (20)
	Non-Hispanic	248 (80)	47 (73)	78 (86)	60 (77)	63 (80)
Race (%)	American Indian/Alaska native	2 (0)	0 (0)	2 (2)	(0)	0 (0)
	Asian	59 (15)	13 (16)	15 (14)	19 (20)	12 (12)
	Black or African American	100 (26)	21 (25)	36 (33)	16 (16)	27 (26)
	Native Hawaiian/Pacific Islander	10 (3)	2 (2)	2 (2)	2 (2)	4 (4)
	Race unknown	89 (23)	26 (31)	16 (15)	23 (24)	24 (23)
	White	132 (34)	21 (25)	37 (34)	37 (38)	37 (36)

^a^Unknown ages = 7

The crude incidence averaged over 4 years was 5.9 per 100,000 population, with a range of 5–6.5 cases/100,000 population. Demographic data by age group and sex for 2020 for Alameda County was not yet available on the US Census Bureau website; hence we could not calculate the age and sex-specific rates for 2020.

**Fig. 2:**
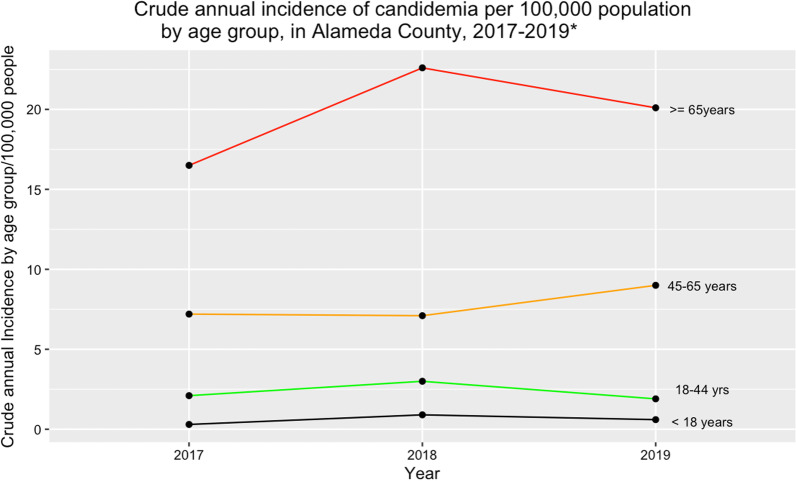
Crude annual incidence of candidemia per 100,000 population by age group, in Alameda County, 2017–2019*. *Alameda County population data for 2020 by sex not yet available on US Census Bureau website

**Fig. 3:**
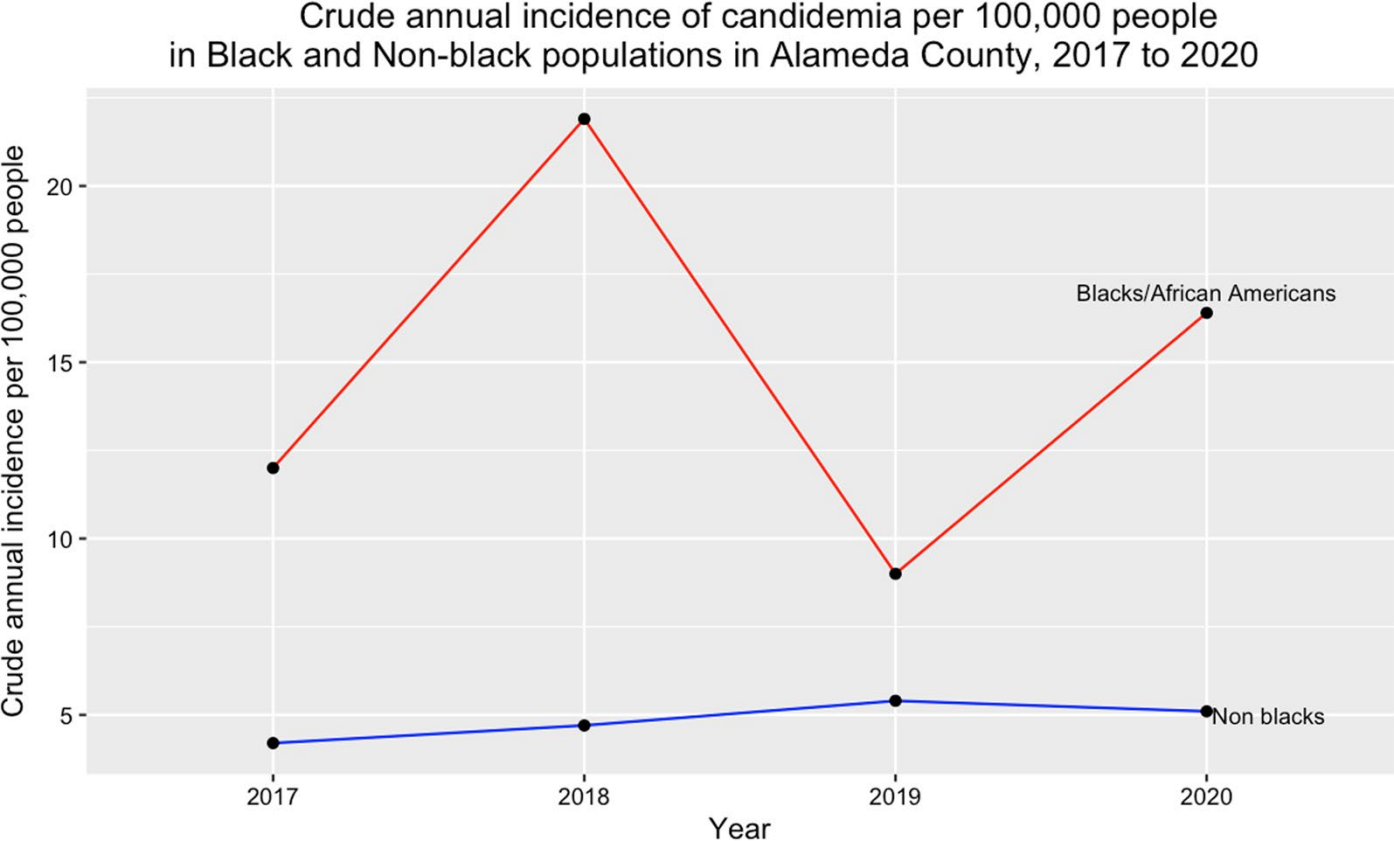
Crude annual incidence of candidemia per 100,000 people in Black and Non-black populations in Alameda County, 2017 to 2020

The crude annual incidence of candidemia, averaged over 3 years (2017–2019) varied by age group. Participants in the ≥ 65-year age group had the highest crude incidence (19.7 per 100,000 population), while those in the < 18 year age group had the lowest annual incidence at 0.6 per 100,000 population. The incidence rate ratio, IRR, comparing the ≥ 18 years of age to the < 18 years was 32.8, p = 0.07. (Fig. 2). The mean crude incidence of candidemia among males from 2017 to 2020 was 6.8 per 100,000 population, which was higher than that for females, 4.8 per 100,000 population. The incidence in Blacks/African Americans averaged 14.8 per 100,000 over the 4-year study period (range: 21.9–9), while for non-blacks, it averaged 4.9 per 100,000 population (range 4.2–5.1). (Fig. 3).

### *Candida* species distribution

**Fig. 4:**
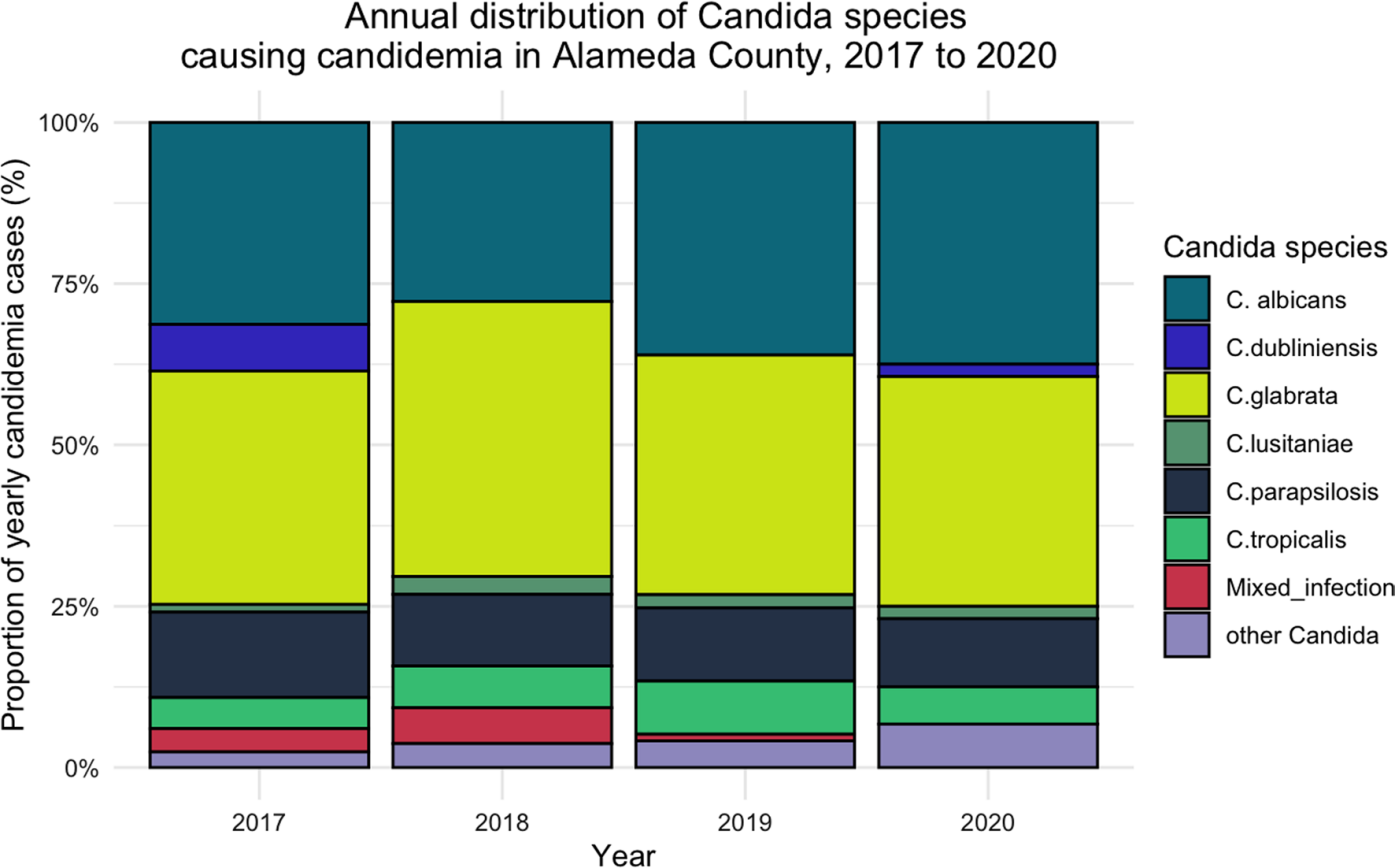
Annual distribution of Candida species causing candidemia in Alameda County, 2017 to 2020

*Candida glabrata* was the most common species, causing candidemia in 149 cases (38.0%). It was followed by *Candida albicans* in 130 cases (33.2%). Though *Candida glabrata* predominated, the difference in the absolute counts between *Candida albicans* and *Candida glabrata* was not significant (p = 0.32, CI − 15.46–5.97). The other species causing candidemia were *Candida parapsilosis*, 45 cases (11.5%), *Candida tropicalis*, 25 cases (6.4%), *Candida dubliniensis*, 8 cases (2.0%), and *Candida lusitaniae*, 8 cases (2.0%). Mixed *Candida* species infections were present in 13 patients (3.3%). Other species of *Candida* accounted for 27 candidemia cases (6.9%) (Fig. 4).

### Underlying conditions and risk factors for candidemia

Most of the patients with candidemia had at least one underlying condition. The most common risk factor present in the candidemia patients was the presence of a central venous catheter. Almost two-thirds (64.3%) of the participants had a central venous catheter in place 2 days before the positive culture (Table 2), while almost half (47.7%) of the patients had been hospitalized in the 90 days before the date of the positive culture. Almost half, (45.9%), of the patients had been present in the ICU during the 14 days before the positive culture.

**Table 2:** Underlying conditions among patients with candidemia, Alameda County, 2017–2020

	Level	Overall	2017	2018	2019	2020
Number		392	83	108	97	104
Previous candidemia (%)	No	382 (97)	83 (100)	104 (96)	91 (94)	104 (100)
	Yes	10 (3)	0 (0)	4 (4)	6 (6)	0 (0)
Hospitalization 90 days before culture (%)	No	187 (48)	33 (40)	46 (43)	56 (58)	52 (50)
	Yes	188 (48)	46 (55)	56 (52)	40 (41)	46 (44)
	Unk	17 (4)	4 (5)	6 (6)	1 (1)	6 (6)
Chronic kidney disease (%)	No	289 (74)	59 (71)	74 (68)	74 (76)	82 (79)
	Yes	103 (26)	24 (29)	34 (32)	23 (24)	22 (21)
Chronic liver disease (%)	No	332 (85)	75 (90)	89 (82)	80 (82)	88 (85)
	Yes	60 (15)	8 (10)	19 (18)	17 (18)	16 (15)
Chronic lung disease (%)	No	292 (74)	68 (82)	77 (71)	66 (68)	81 (78)
	Yes	100 (26)	15 (18)	31 (29)	31 (32)	23 (22)
Diabetes mellitus (%)	No	234 (60)	54 (65)	59 (55)	62 (64)	59 (57)
	Yes	158 (40)	29 (35)	49 (45)	35 (36)	45 (43)
Pregnancy (%)	No	389 (99)	83 (100)	107 (99)	96 (99)	103 (99)
	Yes	3 (1)	0 (0)	1 (1)	1 (1)	1 (1)
Obesity (%)	No	353 (90)	73 (88)	102 (94)	85 (88)	93 (89)
	Yes	39 (10)	10 (12)	6 (6)	12 (12)	11 (11)
Total parenteral nutrition (%)	No	323 (84)	59 (79)	87 (81)	83 (86)	94 (90)
	Yes	60 (16)	16 (21)	20 (19)	14 (14)	10 (10)
Systemic antibiotics 14 days before culture (%)	No	75 (19)	9 (11)	14 (13)	28 (29)	24 (23)
	Yes	311 (81)	71 (89)	91 (87)	69 (71)	80 (77)
Systemic antifungals 14 days before culture (%)	No	285 (73)	17 (20)	95 (90)	83 (86)	90 (87)
	Yes	104 (27)	66 (80)	11 (10)	14 (14)	13 (13)
Central venous catheter (%)	No	139 (36)	25 (30)	34 (32)	37 (38)	43 (41)
	Yes	252 (64)	58 (70)	74 (68)	59 (61)	61 (59)
	Unk	1 (0)	0 (0)	0 (0)	1 (1)	0 (0)
Pancreatitis (%)	No	369 (94)	79 (95)	101 (94)	92 (95)	97 (93)
	Yes	23 (6)	4 (5)	7 (6)	5 (5)	7 (7)
Neutropenia (%)	No	287 (94)	60 (91)	70 (95)	77 (94)	80 (96)
	Yes	18 (6)	6 (9)	4 (5)	5 (6)	3 (4)
ICU admission 14 days before culture (%)	No	206 (53)	42 (52)	61 (58)	47 (49)	56 (54)
	Yes	180 (47)	38 (48)	45 (42)	49 (51)	48 (46)
HIV infection (%)	No	380 (97)	81 (98)	105 (97)	93 (96)	101 (97)
	Yes	12 (3)	2 (2)	3 (3)	4 (4)	3 (3)
Cirrhosis of the liver (%)	No	354 (90)	77 (93)	97 (90)	87 (90)	93 (89)
	Yes	38 (10)	6 (7)	11 (10)	10 (10)	11 (11)
Solid organ transplant (%)	No	390 (100)	82 (99)	108 (100)	97 (100)	103 (99)
	Yes	2 (0)	1 (1)	0 (0)	0 (0)	1 (1)
No surgery 90 days before culture (%)	No	80 (20)	25 (30)	23 (21)	17 (18)	15 (14)
	Yes	312 (80)	58 (70)	85 (79)	80 (82)	89 (86)
Abdominal surgery 90 days before culture (%)	0	341 (87)	64 (77)	95 (88)	89 (92)	93 (89)
	1	51 (13)	19 (23)	13 (12)	8 (8)	11 (11)
Nonabdominal surgery 90 days before culture (%)	No	361 (92)	75 (90)	98 (91)	88 (91)	100 (96)
	Yes	31 (8)	8 (10)	10 (9)	9 (9)	4 (4)
Hematologic malignancy (%)	No	372 (95)	81 (98)	103 (95)	92 (95)	96 (92)
	Yes	20 (5)	2 (2)	5 (5)	5 (5)	8 (8)
Solid organ malignancy (%)	No	356 (91)	71 (86)	97 (90)	89 (92)	99 (95)
	Yes	36 (9)	12 (14)	11 (10)	8 (8)	5 (5)
Metastatic solid organ malignancy (%)	No	351 (90)	73 (88)	97 (90)	88 (91)	93 (89)
	Yes	41 (10)	10 (12)	11 (10)	9 (9)	11 (11)

*Unk* unknown

Overall, 158 patients (40.3%) had diabetes mellitus, which was the most common underlying medical condition in the study participants. Most of the cases 311 (79.3%) had received systemic antibiotics in the 2 weeks preceding the positive *Candida* species culture, while just over one fourth of the cases, 104 (26.5%), had received systemic antifungal medications during the 14 days before the positive culture. Previous candidemia had occurred in just 10 (2.6%) of the incident cases. A few of the cases, 3 (0.8%), were in pregnant women (Table 2).

### Outcome

The case fatality ratio (CFR) 30 days after a positive culture was 36.0%. This CFR also varied by age group; the CFR was 0.7% in the < 18 age group, 6.4% in the 18–44 year age group, 34.8% in the 45–64 year age group and 56.7% in the ≥ 65 years age group. This increase in mortality with increasing age category was significant (p < 0.001).

Case fatality ratios however did not differ significantly by race. Comparing Blacks with non-blacks, 36 of 100 black participants died (36.0%), and 105 of 292 (35.9%) of non-blacks died (p = 0.99). In addition, the CFR did not vary by sex. Approximately one-third of the male patients, 84/229 (36.7%) died, as did a similar proportion of female patients, 57/163 (35.0%) (p = 0.81).

### Predictors of mortality

**Fig. 5:**
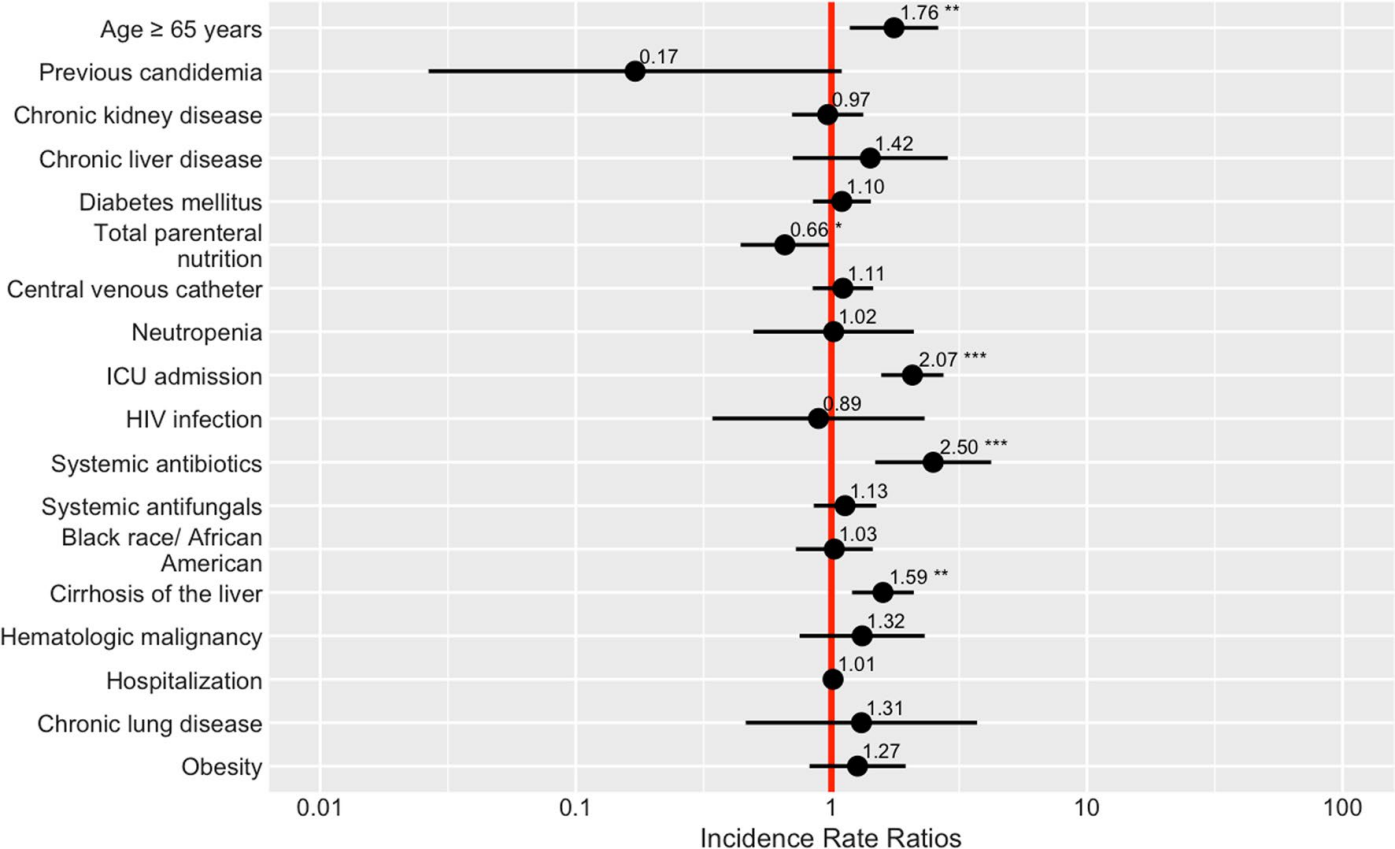
Summary of unadjusted bivariate analysis of selected risk factors for mortality among candidemia patients in Alameda County, 2017–2020

Bivariate regression models showed several risk factors for mortality among patients with candidemia including age ≥ 65 years, total parenteral nutrition, chronic liver disease, prior systemic antibiotic therapy, cirrhosis of the liver, and prior ICU admission before culture being significant predictors of mortality (Fig. 5). However, on multivariate analysis, just age ≥ 65 years (RR 2.01, CI 1.51–2.68, p < 0.001), prior receipt of systemic antibiotic therapy, (RR 1.84, CI 1.06–3.17, p = 0.03), cirrhosis of the liver (RR 1.82, CI 1.36–2.43, p < 0.001), and prior admission to the ICU before culture (RR1.66, CI 1.28–2.14, p < 0.001) remained significant predictors of mortality (Table 3). Mortality did not differ by *Candida* species (X^2^ = 12, p = 0.09).

**Table 3:** Predictors of mortality among candidemia patients in Alameda County, 2017–2020

Characteristic	RR	95% CI	p-value
ICU admission before culture	1.66	1.28, 2.14	< 0.001
Cirrhosis of the liver	1.82	1.36, 2.43	< 0.001
Systemic antibiotic therapy	1.84	1.06, 3.17	0.029
Age ≥ 65 years	2.01	1.51, 2.68	< 0.001

*RR* risk ratio, *CI* confidence interval

## Discussion

Our results highlight that NAC species accounted for the majority of candidemia cases in Alameda County over the 4-year study interval from 2017 to 2020. During that time, *Candida glabrata* was becoming the dominant *Candida* species implicated in candidemia. Our results also reaffirmed the well-established finding that candidemia is an opportunistic infection [17–19]. The CFR was high (36.0%), and the significant independent predictors of mortality in our patients were age ≥ 65 years, ICU admission, cirrhosis of the liver, systemic antibiotic therapy, and prior ICU admission.

Our findings concerning the distribution of *Candida* species differ from results of other studies [10–12] that showed that *Candida albicans* was still the dominant species causing candidemia. However, our results are similar to those reported by Ma et al., based on a review of candidemia cases in a tertiary care hospital in China, which showed a NAC, *C. tropicalis*, to be the leading cause of candidemia [6]. Furthermore, our results are consistent with findings from the 15-year FUNGINOS Survey in Switzerland, which illustrated a significant decrease, from 60 to 53% (p = 0.0023) in the proportion of cases of candidemia caused by *C. albicans* and an increase (18% to 27%) in the fraction of cases due to *C. glabrata* [9] from 2004 to 2018. Other studies have reported similar trends [9, 20–22]. Non-*Candida albicans* species, which are usually more drug resistant, were responsible for 66.0% of candidemia cases in our study, similar to the findings of other studies that reported proportions of NAC of over 60.0% [12, 23]. This increase in NAC could be explained by the increased use over the last two decades of azole antifungal drugs, which exert selection pressure on *Candida* spp. and favor resistant organisms, like *Candida glabrata*. Multiple other reasons may also explain the differences in the distribution of *Candida* spp. involved in candidemia, including geographical and ecological factors; variability in monitoring and reporting systems; characteristics of the patient populations; and infection prevention and control strategies [24].

The crude cumulative annual incidence of candidemia in our population was 5.9 per 100,000 population (range 5–6.5) and was slightly lower than the 8.7 per 100,000 population reported by Toda et al., when averaging the incidence rates across four sites in the USA between 2012 and 2016 [12]. The incidence of candidemia in our study did not vary by sex. The average crude cumulative incidence among males, (6.8 per 100,000 population), was 1.4 times that in females (4.8 per 100,000 population) over the same period, similar to findings reported by Toda et al. [12]. Increasing age was significantly associated with an increasing crude cumulative incidence. After stratifying our population into those less than 18 years and ≥ 18 years of age, the average cumulative incidences were 0.6 per 100,000 population and 19.7 per 100,000 population, respectively. The incidence rate ratio, IRR, comparing the ≥ 18 years of age to the < 18 years was 32.8 (p = 0.07). This difference could be explained by the higher prevalence of underlying conditions which predispose to candidemia in the older age group.

The crude cumulative incidence amongst Blacks was higher than that amongst nonblacks, 14.8 per 100,000 vs 4.9 per 100,000 population. Similar findings have been reported by others [12, 23]. The difference in candidemia incidence by race might be a proxy for socioeconomic disparities between blacks and non-blacks which play a role in the disparities in the prevalence of underlying conditions between Blacks and non-blacks [25, 26].

The risk factors for candidemia in our study were consistent with what has been reported previously [6, 13, 17, 19, 27]. Routes of entry (e.g., a central venous catheter, surgical wound) and the immunosuppression associated with certain diseases, such as HIV infection, diabetes mellitus, and chronic kidney disease, favor the introduction and proliferation of *Candida* spp. in the blood.

Mortality was high in our study population, with a case fatality ratio of 36.0%, slightly lower than that reported in other studies [12, 18, 23], which have reported CFR’s of about 50.0%. This difference is likely due to differences in the study populations, as some of the earlier studies were done in ICUs and tertiary hospitals, which generally have sicker patients.

The findings of increasing age and cirrhosis of the liver being independent significant predictors of mortality among patients with candidemia are consistent with findings from a different study [27]. Our finding of prior receipt of systemic antibiotic therapy being a significant predictor of mortality in our study could have been a proxy for sepsis or sepsis-related organ failure in some patients. Contrary to another study [6], we did not find the presence of a central venous catheter to be a significant predictor of mortality among patients with candidemia, perhaps because of improved catheter care in recent years, thus reducing the risks associated with infection by other micro-organisms and superinfection with *Candida* spp.

## Limitations

Our study has several limitations. First, the data were collected only from Alameda County, which has a Mediterranean climate, and a higher median household income than the general US population; thus, the results might not be readily generalizable to the entire US population. Second, only 4 years were assessed which was not sufficient for us to examine moderate to long term temporal trends. Thirdly, a considerable proportion (22.7%) of our study participants do not have their race data specified, and this introduced some information bias (misclassification) in our race-specific analysis, the direction of which is unknown.

However, the data used in this analysis were collected from a demographically diverse population by an active surveillance program, CEIP, which captures 100% of its catchment area.

## Conclusion

Non-albicans *Candida* species currently account for the majority of candidemia cases in Alameda County. Black populations are disproportionately affected by this opportunistic infection and mortality in candidemia patients remains high. It is important to continue surveillance for candidemia so that empiric antifungal treatments can better target likely causative organisms.

## Supplementary Information

**Additional file 1.** Candidemia dataset csv.

**Additional file 2.** Data dictionary, candidemia csv.

## Data Availability

All data generated or analysed during this study are included in this published article and its Additional files.
