# Supplementary material for: Epidemiologic features, clinical characteristics, and predictors of mortality in patients with candidemia in Alameda County, California; a 2017–2020 retrospective analysis
Source: BMC Infect Dis. 2022 Nov 12;22:843. doi: 10.1186/s12879-022-07848-8 (PMC9652840; doi:10.1186/s12879-022-07848-8)
Supplement: Supplementary file 2 — Additional file 2. Data dictionary, candidemia csv. [file 12879_2022_7848_MOESM2_ESM.pdf]

DESCRIPTION OF THE CANDIDEMIA DATASET

This is a description of the main variables that are relevant to our analysis of candidemia patients in this study. The dataset found here is the cleaned dataset which contains only the unique rows of data which ended up being relevant to our study. A brief description of the main variables is in the table below:

Number	Variable name	Description
1	patientid	Unique patient identifier
2	year	Year in which patient had candidemia episode
3	reportdt	Date candidemia case was reported to the CEIP
4	prevcandidemia	Patient's history of previous candidemia episode<br>0 – No history 1, Positive history
5	initcxdt	Initial blood culture date
6	calcageyyyy	Patient's calculated age in years
7	sex___m	Male sex: 0- No, 1- Yes
8	sex___f	Female sex: 0 -No, 1- Yes
9	sex___t	Transgender: 0- No, 1 - Yes
10	race___white	White race: 0 – no, 1 – yes
11	race___black	Black/African American race: 0 – no, 1 – yes
12	race___asian	Asian race: 0 – no, 1 – yes
13	race___nativehawaiian	Native Hawaiian or Pacific Islander race: 0 – no, 1 – yes
14	race___amerindian	Native or American Indian race: 0 – no, 1 – yes
15	race___unk	Unknown race: 0 – no, 1 – yes
16	ethnicity	Ethnicity: Hispanic, Non- Hispanic, N/A -unknown
17	cxsource___cvc	Central venous catheter blood culture source:<br>0 – no, 1 – yes
18	cxsource___periph	Peripheral catheter blood culture source<br>0 – no, 1 – yes
19	cxsource___unspecbld	Unspecified blood culture source<br>0 – no, 1 – yes
20	cxsource___oth	Other culture source<br>0 – no, 1 – yes
21	cxsource___unk	Culture source unknown<br>0 – no, 1 – yes
22	addlorgs	Additional organisms present in blood culture other than Candida sp 0- no, 1- yes
23	subqposcx	Subsequent positive culture<br>0 – no, 1 – yes

24	hosp	Was the patient hospitalized on the day of or the 6 days after the date of initial specimen collection?<br>0 – no, 1 – yes
25	admdt	Admit date
26	preadmloc	Pre-admission location; LTCF- Long term care facility, hosp inpat- hospitalized inpatient, private res- private residence, homeless, other
27	outcome	Outcome: 1- Death, 0 - alive
28	hosp90dbeforecx	Hospitalized 90 days before positive culture: 1-yes, 0 - no
29	underlycond___none	No underlying condition: 1- yes, 0 - no
30	underlycond___kidn	Renal underlying condition: 1 – yes, 0 -no
31	underlycond___liver	Chronic liver disease<br>0 – no, 1 – yes
32	underlycond___cpd	Chronic pulmonary disease<br>0 – no, 1 – yes
33	underlycond___dm	Diabetis mellitus<br>0 – no, 1 – yes
34	underlycond___pregnant	Pregnant?<br>0 – no, 1 – yes
35	underlycond___obesity	Obesity<br>0 – no, 1 – yes
36	tpn	Did the patient receive total parenteral nutrition (TPN) in the 14days before, not including the date of initial specimen collection?<br>0 – no, 1 – yes
37	sysantibact	Systemic antibiotic administration in the two weeks before positive culture (including date of initial specimen collection)<br>0 – no, 1 – yes
38	curaf	Systemic antifungal administration in the two weeks before positive culture (including date of initial specimen collection)<br>0 – no, 1 – yes
39	cvc	Did the patient have a CVC in the 2 calendar days before, not including the date of initial specimen collection<br>0 – no, 1 – yes

40	pancreatitis	Pancreatitis in the 90 days before, not including the date of initial specimen collection<br>0 – no, 1 – yes
41	neutropenia	Was the patient neutropenic in the 2 calendar days before, not including the date of initial specimen collection?<br>0 – no, 1 – yes
42	icubeforecx	Was the patient in the ICU in the 14 days before the date of initial specimen collection?<br>0 – no, 1 – yes
43	icuaftercx	Was the patient in an ICU on the day of incident specimen collection or in the 13 days after the date of initial specimen collection?<br>0 – no, 1 – yes
44	aids	AIDS<br>0 – no, 1 – yes
45	underlycond___hiv	HIV positive<br>0 – no, 1 – yes
46	Liverdis___cirr	Cirrhotic liver disease<br>0 – no, 1 – yes
47	underlycond___transcell	Hematopoietic/ Stem cell transplant<br>0 – no, 1 – yes
48	underlycond___transorg	Solid organ transplant<br>0 – no, 1 – yes
49	surg___abdom	Abdominal surgery 90 days before positive culture<br>0 – no, 1 – yes
50	surg___nonabdom	Non-abdominal surgery 90 days before positive culture<br>0 – no, 1 – yes
51	surg___none	No surgery 90 days before positive culture<br>0 – no, 1 – yes
52	underlycond___hemat	Hematologic malignancy<br>0 – no, 1 – yes
53	underlycond___nonmeta	Nonmetastatic solid organ malignancy<br>0 – no, 1 – yes
54	underlycond___meta	Metastatic solid organ malignancy<br>0 – no, 1 – yes
55	species___ca	C. albicans
56	species___cg	C. glabrata
57	species___cp	C. parapsilosis
58	species___ct	C. tropicalis
59	species___cd	C. dubliniensis
60	species___cl	C. lusitaniae

61	species___ck	C. krusei
62	species___cgm	C. guilliermondi
63	species___co	Candida, other
64	species___cgn	gram tube negative/non-albicans
65	Candida_Yes	Mixed Candida infection or not<br>2 – yes, 1 – no,
66	Mixed_infection	Presence of more than one Candida species<br>0 – no, 1 – yes
67	Species_ck_cgm_cgn_co	Presence of either Candida krusei or C. guilliermondii or Candida, gram tube negative/non-albicans, or Candida, other:<br>0 – no, 1 – yes
68	Age_grouped	Age groups
69	sex	Sex: 0 - Male, 1 - Female
70	race	Race: 0 – not black/African American, 1 – Black/ African American
71	Candida_grouped	Different species of candida in longitudinal fashion
72	Race_grouped	Races arranged in longitudinal fashion
73	Black_race_or_not	Patient is of black race or not<br>0 – not black, 1 - black
74	Age_new	Age 65 years and older:<br>0 – no , 1 – yes
